# Determinants of Cause-Specific Mortality and Loss of Independence in Older Patients following Hospitalization for COVID-19: The GeroCovid Outcomes Study

**DOI:** 10.3390/jcm11195578

**Published:** 2022-09-22

**Authors:** Chukwuma Okoye, Valeria Calsolaro, Alessia Maria Calabrese, Sonia Zotti, Massimiliano Fedecostante, Stefano Volpato, Stefano Fumagalli, Antonio Cherubini, Raffaele Antonelli Incalzi, Fabio Monzani

**Affiliations:** 1 Geriatrics Unit, Department of Clinical and Experimental Medicine, University of Pisa, Via Savi 10, 56126 Pisa, Italy; 2 Aging Research Center, Department of Neurobiology, Care Sciences and Society, Karolinska Institutet and Stockholm University, 17165 Stockholm, Sweden; 3 Geriatrics Unit, Department of Medicine, Campus Bio-Medico University and Teaching Hospital, 00128 Rome, Italy; 4 Geriatria, Accettazione Geriatrica e Centro di Ricerca per l’Invecchiamento, IRCCS INRCA, 60124 Ancona, Italy; 5 Department of Medical Sciences, University of Ferrara, 44121 Ferrara, Italy; 6 Geriatric Intensive Care Unit, Department of Experimental and Clinical Medicine, University of Florence, 50121 Firenze, Italy

**Keywords:** COVID-19, follow-up, disability, functional outcome, long COVID

## Abstract

Hospitalization for acute SARS-CoV-2 infection confers an almost five-fold higher risk of post-discharge, all-cause mortality compared to controls from the general population. A negative impact on the functional autonomy of older patients, especially in cases of severe disease and prolonged hospitalization, has been recently described. However, little is known about the determinants of cause-specific mortality and loss of independence (LOI) in the activities of daily living (ADL) following COVID-19 hospitalization. Thus, the current prospective, multicenter study is aimed at identifying the determinants of post-discharge cause-specific mortality and the loss of autonomy in at least one ADL function. Older patients hospitalized for a SARS-CoV-2 infection were consecutively enrolled in an e-Registry from 1 March 2020, until 31 December 2020. After at least six months from discharge, patients were extensively re-evaluated according to a common protocol at the outpatient clinic of eight tertiary care Italian hospitals. Of 193 patients [109 (56.4%) men, mean age 79.9 ± 9.1 years], 43 (22.3%) died during follow-up. The most common causes of death were cardiovascular diseases (46.0%), respiratory failure (26.5%), and gastrointestinal and genitourinary diseases (8.8% each). Pre-morbid ADLs qualified as an independent mortality risk factor [adjusted HR 0.77 (95%CI: 0.63–0.95)]. Of 132 patients, 28 (21.2%) lost their independence in at least one ADL. The adjusted risk of LOI declined with a lower frailty degree [aOR 0.03 (95%CI: 0.01–0.32)]. In conclusion, at long-term follow-up after hospitalization for acute SARS-CoV-2 infection, more than 40% of older patients died or experienced a loss of functional independence compared to their pre-morbid condition. Given its high prevalence, the loss of functional independence after hospitalization for COVID-19 could be reasonably included among the features of the “Long COVID-19 syndrome” of older patients.

## 1. Introduction

In March 2020, the SARS-CoV-2 pandemic was declared by the World Health Organization (WHO); since then, more than 230 million cases and almost 5 million deaths have been reported worldwide [1]. The scientific community has widely studied the spreading of the disease and the acute clinical consequences, affecting the respiratory system with pneumonia, respiratory failure, and several other organs, with different symptoms and severity [2]. The long-term consequences of SARS-CoV-2 infection have gained more significant interest in the last few months. COVID-19-related symptoms after the acute phase of the disease have been reported in various amounts to define the “post-acute COVID-19 syndrome” or “Long Covid” [3]. Unfortunately, this vast amount of data does not sufficiently include the oldest old patients, who massively suffered from COVID-19 and its sequelae. Interestingly, Bhaskaran et al. [4] reported that patients discharged from COVID-19 hospitalization showed an almost five-fold higher risk of all-cause mortality than controls from the general population.

Although various studies reported chronological age as a risk factor for rehospitalization and mortality among COVID-19 survivors [5,6,7], no investigation evaluated case-specific death rates of older patients. Moreover, a principal goal of the care of older patients is preserving the ability to perform basic self-care activities, which are fundamental to maintaining older people′s independence and quality of life. Indeed, loss of autonomy in basic activities, known as activities of daily living (ADL), is strongly associated with institutionalization and death [8]. In this setting, a recent study showed that hospitalization for acute SARS-CoV-2 infection harmed older patients’ functional autonomy, especially in those with severe disease and more impaired basal ADLs [9]. Nonetheless, albeit even the loss of a single ADL function is associated with an increased likelihood of requiring long-term nursing care [10] and poor outcomes [11], far too little attention has been devoted to the loss of functionality after hospitalization for COVID-19. Thus, none of the pre-existing literature evaluated the determinants of cause-specific mortality and loss of independence through an extensive follow-up, from hospital admission to long-term outpatient clinic re-evaluation. Given these premises, the current multicenter, prospective study aimed at evaluating the determinants of long-term, cause-specific mortality and loss of independence (defined as the loss of at least 1 ADL at the outpatient clinic visit) in older patients following hospitalization for COVID-19.

## 2. Materials and Methods

### 2.1. Study Population

The GeroCovid Observational is a multi-purpose and multicenter registry endorsed by the Italian Society of Gerontology and Geriatrics in collaboration with Blue Companion that aims at investigating the impact of SARS-CoV-2 pandemics on older patients in different settings of care. The objectives of the project are setting specific. In GeroCovid Acute Wards Study, individuals aged 60 years or older, hospitalized for SARS-CoV-2 infection, were consecutively enrolled in an e-Registry from 1 March 2020 until 31 December 2020. Before hospital discharge, enrolled patients were asked to consent to participate further in the GeroCovid Outcomes Follow-up Study. Therefore, all the inpatients able to give informed consent were prospectively studied without exclusion criteria.

Eight investigational sites of tertiary care Italian hospitals participated in the outpatient follow-up. The follow-up protocol and assessments have been published previously [12]. The study is registered on clinicaltrial.gov (Trial Registration: NCT04379440; accessed on 3 June 2022).

### 2.2. Data Collection

At hospital admission, GeroCovid researchers collected data regarding the demographic characteristics (sex, age, race), living arrangements, smoking habits, and pre-COVID-19 mobility (categorized as moving independently, using walking aid/moving with a wheelchair, moving with assistance in a wheelchair/bedridden). Moreover, the patients were asked to report whether they could independently perform (defined as not needing the assistance of another person) each of the six basal ADLs (toileting, bathing, transferring, eating, dressing, and walking across a small room) [8]. We defined ADL dependence as the self-report of being unable to perform an ADL or requiring the help of another person for each ADL at baseline and follow-up. The outcome was a 0 to 6 scale of the functional autonomies in ADL, with a score of 6 representing the independence in all the ADLs [13].

The presence of the following chronic diseases was derived from medical records: systemic arterial hypertension, cardiovascular diseases (including cardiomyopathies, ischemic heart disease, heart failure, atrial fibrillation), chronic obstructive pulmonary disease (COPD), diabetes mellitus, obesity, chronic renal failure, depression, and dementia.

Regarding COVID-19 severity during hospitalization, we divided participants’ clinical status at the beginning and the end of hospitalization into four clinical categories’ ordinal scale according to the classification recommended by the WHO R&D Blueprint expert group [14]: (1) patients not requiring oxygen therapy; (2) patients requiring oxygen by mask or nasal prongs. (3) with high-flow oxygen or non-invasive ventilation (HF/NIV); (4) needing intubation and mechanical ventilation. The severity of respiratory failure was assessed by calculating the PaO2/FIO2 ratio [15] (i.e., partial pressure arterial oxygen/fraction of inspired oxygen ratio) of the first arterial blood gas analysis performed at ward admission (in the case of using Venturi mask, the FIO2 indicated in the swivel connector was utilized). Among biochemical parameters, we considered the following inflammatory markers routinely assessed at ward admission: total white blood cells (WBC) count, neutrophils and lymphocytes amount, neutrophils/lymphocytes ratio (NLR), and C-reactive protein (CRP) value.

As an indirect measurement of frailty, we calculated a 30-item Frailty Index (FI) score in each patient, as Searle et al. [16], taking into account a wide range of signs, symptoms, disabilities, and diseases before hospital admission. Each item of the FI was scored as ‘0′ (absence of the deficit) or ‘1′ (presence of the deficit). The FI was calculated as the ratio between the number of health deficits of the individual and the total number of health items considered for its computation (*n* = 30, see Table S1 in the Supplementary Materials). We then categorized the FI as follows: >0.3 frail; between 0.08 and 0.3 pre-frail; <0.08 robust [16].

### 2.3. Ambulatory Follow-up

Six months after hospital discharge, surviving patients were clinically re-evaluated through physical examination and CGA whenever possible or interviewed by phone. Due to the COVID-19 s pandemic wave overlapping and the subsequent National lockdown, 20% of the follow-up visits were done within eight months and 7% within nine months from hospital discharge. During the ambulatory visit, all patients underwent physical examination and a comprehensive geriatric assessment, including a minimum dataset of the following scales: ADL [17] and IADL [18], Cumulative Illness Rating Scale (CIRS) [19], and Mini-Mental Status Examination (MMSE) [20]. At the 6-month follow-up, failure to perform an ADL not previously affected without assistance was considered a new ADL deficiency or loss of independence [8,13]. Death causes were asked by phone call directly to the caregiver or patients’ familial and confirmed through a Health Regional computerized archive.

### 2.4. Statistical Analysis

Statistical analysis was performed by IBM SPSS Statistic (IBM SPSS Statistic version 27.0 lnk IBM Corporation and its licensor 1989–2020). Continuous variables were presented as mean and standard deviation, ordinal variables as the median and interquartile range (IQR), and categorical variables as a percentage. Mann–Whitney U-test and chi-square test were used for comparisons between survived and deceased patients and between patients with at least one ADL loss (AL) vs. those without ADL loss (non-AL). The risk of mortality was evaluated using a Kaplan Meier estimator. After checking the proportional hazards assumption using Schöenfeld residuals, the hazard ratio (HR) and 95% confidence interval (95%CI) of mortality were calculated for each demographic and clinical record collected at the time of in-hospital enrolment. For multivariate analysis, besides age and sex, model covariates were *a priori* selected as follows: history of heart failure, coronary artery disease, hypertension, diabetes mellitus, obesity, chronic renal disease prior to stroke, or transient ischemic attack. After adjusting for potential confounders, the hazard ratio of long-term mortality was calculated.

In the comparison between patients with and without AL, severely disabled patients (premorbid BADL ≤1) were excluded from the analysis. To evaluate the strength of association among each ADL lost, a Cramer-V coefficient was assessed using a matrix correlation: a Cramer-V coefficient ranging from 0.30 and 0.50 indicated low-to-moderate correlation, from 0.51 and 0.70 a moderate-to-high correlation and, values >0.70 a very strong correlation. Finally, multivariate logistic regression was performed to determine LOI risk factors using demographic and clinical covariates recorded at admission as independent variables and LOI as the dependent variable. Estimate odds ratios (ORs) with 95% confidence intervals (CIs) were obtained using the same covariates selected for multivariate analyses. Tests were performed considering a level of significance of 5%.

## 3. Results

One hundred ninety-three patients [109 (56.4%) men, mean age 79.9 ± 9.1 years] were enrolled in the study. At baseline, 122 (63.8%) inpatients were able to walk independently, 12.5% walked with a walker, and 8.4% needed a cane to walk. According to WHO status, most patients (53.4%) were admitted to wards with moderate disease, 23.4% with mild disease, 21.8% with severe disease, and 3.6% with critical disease. The median follow-up time was 6.5 months (range 5.4–8.8), and 43 patients died with a mean survival time of 7.9 months (Figure S1). The most common causes of death were cardiovascular disease (46.0%), respiratory failure (26.5%), and genitourinary organ-related disease (8.8%) (Table S2). Clinical differences between survivors and non-survivors are shown in Table 1.

Compared to survivors, deceased patients were older, more likely women, more disabled, and frailer. No relevant differences were observed concerning inflammatory markers or degree of respiratory failure between survived and deceased patients. Similarly, no difference was found in terms of COVID-19 severity, length of stay, and prevalence of comorbidities. Moreover, as shown in Table S4, drug therapy during hospital stay did not correlate with long-term prognosis in our cohort. More in-depth, 113 (60.4%) patients received antibiotics, 47 (25.1%) antiviral drugs (lopinavir/ritonavir or remdesivir), 127 (67.9%) Low-weight molecular heparin, 9 (4.8%) immune-modulators drugs (i.e., Baricitinib or Tocilizumab), 89 (47.6%) patients received intravenous corticosteroids, 62 (33.2%) patients hydroxychloroquine.

**Figure 1:**
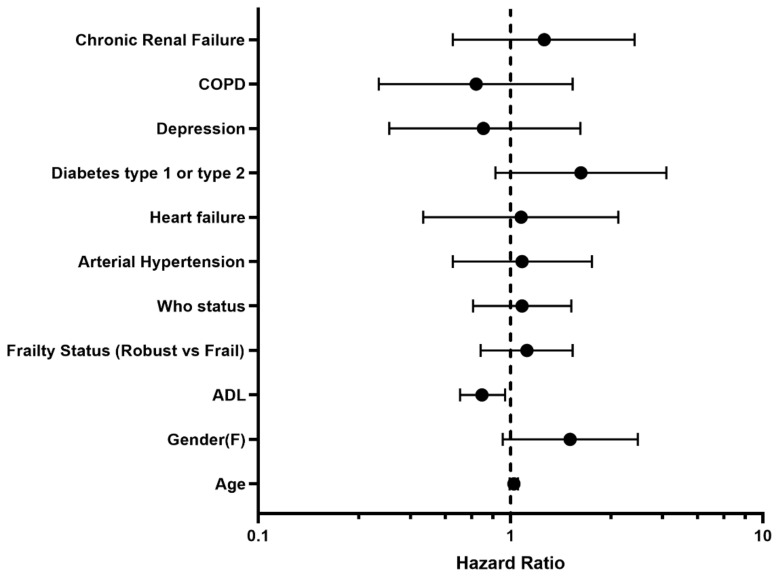
Determinants of six-month post-discharge all-cause mortality. COPD: chronic obstructive pulmonary disease; WHO: world health organization; ADL: activities of daily living.

By univariate Cox regression, type 2 diabetes mellitus (crude HR 2.33, 95%CI: 1.20–4.54) and decreasing ADL (crude HR 0.68; CI: 0.59–0.79) emerged as significantly associated with all-cause mortality whereas, after adjustment for potential confounders, the adjusted mortality risk declined with increasing pre-morbid ADL (adjusted HR 0.77; 95%CI: 0.63–0.95) (Figure 1).

Two patients were lost at the six-months follow-up, and 16 patients were excluded from the analysis due to severe disability prior to hospital admission (BADL ≤ 1). As shown in Table 2, out of the remaining 132 patients, 28 had at least one ADL loss (AL) at follow-up visit. Bathing (29%), continence (16%), toileting (16%), and transferring (13%) were the most common basic activities lost. Patients frequently experienced the loss of more ADL, and the loss of bathing autonomy frequently coexisted with that of dressing (Cramer’s v = 0.902); nevertheless, we found a significant correlation in each pairwise comparison between each ADL and another (*p* < 0.001, see Table S3 in the Supplementary Materials).

**Figure 2:**
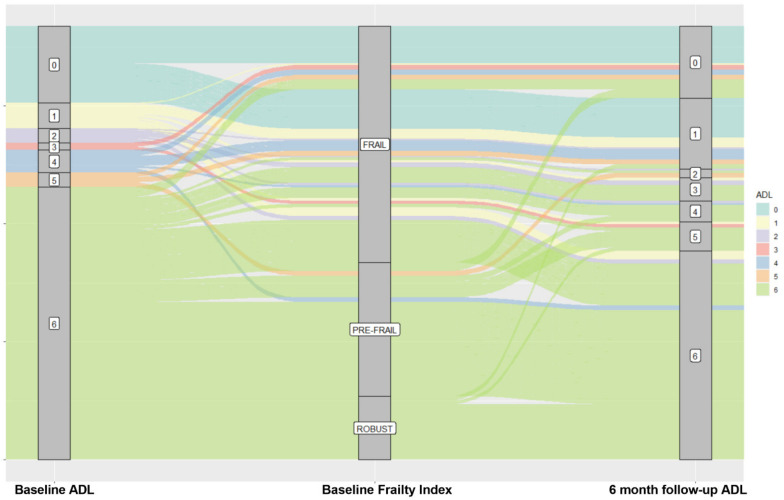
Modifications of ADL according to Frailty Status at 6-month follow-up. Alluvial Plot.

As shown in Table 2, patients with AL were more likely to be frail as compared to their counterparts (see Figure 2), more likely to be females, experiencing a longer hospital stay, and with a higher number of comorbidities. No relevant differences between AL and non-AL patients were found in terms of P/F ratio, neutrophils/lymphocyte ratio, and Hs-CPR at nadir.

The figure shows how frailty status strongly influences the loss of ADL. Robust patients (at baseline evaluation) patients were less likely to experience loss of ADL at 6 month-follow up; conversely, the majority of pre-frail and frail patients showed a downward shift of ADL after 6 months.

**Figure 3:**
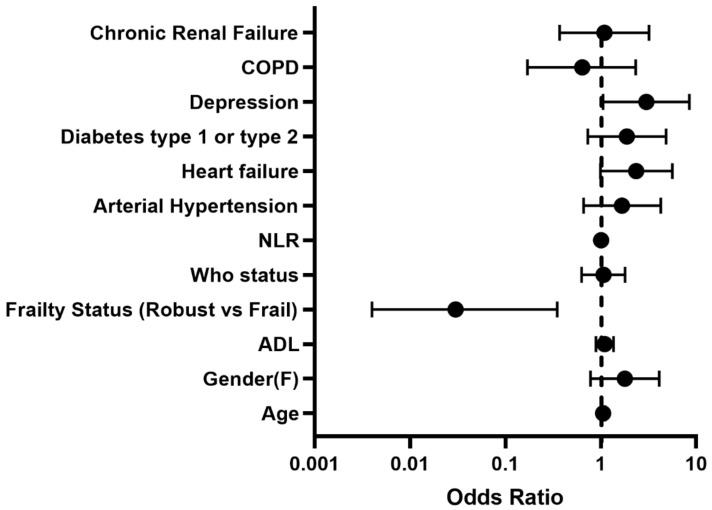
Determinants of six-month loss of independence. COPD: chronic obstructive pulmonary disease; NLR: neutrophils/lymphocytes ratio; WHO: world health organization; ADL: activities of daily living.

Furthermore, AL patients were almost two-fold more likely to have a history of depression than controls (25% vs. 10%, *p* = 0.033). At multivariable logistic analysis, the adjusted risk of LOI declined with higher frailty status (robust status–crude OR: 0.04, 95%CI 0.01–0.34, adjusted OR 0.04, 95%CI 0.01–0.35), while depression failed to reach statistical significance after adjusting for confounders (crude OR 3.00, 95CI% 1.05–8.51, adjusted OR 2.93, 95CI%; 0.94–9.09, (see Figure 3).

## 4. Discussion

In our multicentet longitudinal study, we found that after 6-month hospital discharge for COVID-19, more than 40% of older patients were deceased or encountered a loss of independence compared to their pre-morbid condition. A higher level of functional dependence, defined as needing human help with ADLs, persisted as an independent mortality risk factor after extensive adjustment for other comorbidities; moreover, the frailty degree independently predicts the risk of disability onset.

Our comprehensive study found a 22% six-month mortality rate which is significantly greater than the 1% reported in the Romero-Duarte study [3] on a younger population (mean age 63.0 years). On the contrary, we found a 7% lower 180-day post-discharge mortality compared to Gunster et al. [5] study; yet in this latter study, the enrolled population (mean age 72 years) included a relevant proportion of ventilated patients that expressed up to 71% six-month mortality. In contrast, only 35.1% of our patients received ventilatory support or high concentration Oxygen (i.e., NIV or high-flow oxygen therapy or Oxygen therapy through Venturi mask at FiO2 > 40%). Those who survived were younger, more likely to be men, and with higher functional independence than deceased patients. However, after multiple adjustments for confounders, only pre-admission ADLs qualified as an independent mortality predictor, consistent with Carrillo-Garcia et al.’s study, demonstrating a 5-fold higher 3-month post-discharge mortality in older patients hospitalized for COVID-19 with severe dependence (BI < 40) than their counterparts [21]. Interestingly, both the level of inflammatory markers at admission and the degree of disease severity did not emerge as mortality risk factors in our study, at odds with Jovanoski et al. [22] reporting a significant relationship between severe COVID-19 and poor outcomes. At least two reasons might account for this discrepancy: firstly, survivors who experienced a critical COVID-19 were more likely to be younger (and less frail) than counterparts (71.2 vs. 80.2 years, respectively, data not shown), thus explaining a faster recovery and a more favorable outcome after the acute illness than older peers. Secondly, COVID-19 has a highly variable disease course within the hospital stay [23]; therefore, clinical severity and circulating levels of HS-CRP or leukocyte count at admission could not reflect the actual course of the illness during hospitalization.

To our knowledge, there are insufficient data regarding death causes among older COVID-19 survivors. In our study, most cases of 6-month overall mortality were due to cardiovascular disorders, respiratory and urinary tract infections, in partial disagreement with Bhaskaran et al. study [4], reporting cancer, circulatory disease, and other respiratory causes as leading causes of death after COVID-19 hospitalization. However, in the afore-mentioned study, data were collected from ICD-10 codes, with a tendency of the clinician to code COVID-19 for a range of clinical complications, masking more specific sequelae. In contrast, in our study, death causes were asked by phone directly to the caregiver or patients’ familial and subsequently checked through Health Regional computerized archives. We observed increased rates of respiratory failure in our cohort of post-COVID older patients, thus indicating the well-established long-lasting lung impairment caused by the virus [24]. Moreover, among patients deceased due to acute cardiac diseases, heart failure, and acute myocardial injury were the most reported death diagnoses, further confirming the detrimental COVID-19 effect on the cardiovascular system by exacerbating heart failure in patients with pre-existing comorbidities or through myocardial inflammatory involvement, regardless disease severity [25].

It is commonly assumed that regardless of COVID-19 severity, a vast proportion of patients experience symptoms after the acute phase of the disease in the so-called post-acute COVID-19 syndrome or “Long COVID” [3]. Nevertheless, most studies have not explicitly dealt with the loss of functionality in older patients. Data from a large population-based cohort [26] reported higher odds of worsening mobility in non-hospitalized adults with COVID-19 compared with those without COVID-19, suggesting policymakers’ appropriate interventions also for individuals with mild to moderate disease. In our study, more than one-fifth of the patients lost at least one ADL, bathing, dressing, and transferring the most common ones, probably due to increased difficulty in completing tasks requiring balance and coordination. As shown in Figure 2, most study participants were non-frail at hospital admission. They had a median ADLs score of 6/6, thus underlying our cohort’s functional “robustness” prior to hospitalization (See Figure 3). In older patients with COVID-19, rather than disease severity, the effect of prolonged immobilization due to extended hospital stay, reduced nutritional intake, and isolation could further trigger the occurrence of new disability. Notably, patients losing ADL had a significantly more extended hospital stay than their counterparts, in agreement with a recent study by Gómez-Uranga et al. [9], demonstrating how frailty is related to longer hospitalization for COVID-19, harming nutritional and functional status following hospital discharge. Accordingly, in the present study, we observed the presence of a persistent functional disability in one-fifth of patients following hospital discharge, thus underlying the possibility of including the functional loss in the definition of the Long COVID symptoms in older adults.

Concerning comorbidities, we found no relevant differences between AL and controls. However, it is worth noting that patients with a diagnosis of depression were two-fold more likely to lose ADLs as compared to patients without depression. Indeed, hospitalization, social isolation, and medications may have exacerbated depression symptoms in these high-risk patients, thus worsening their functional status.

Compared to our results, a study by Ferrante et al. [27] showed similar trajectories of functionality loss among older patients (mean age 83.3 years) following critical illness in a non-COVID Intensive Care Unit (ICU), typically in those who had high levels of pre-morbid disability. Conversely, Andrew et al. [28] found only an 8.2% moderate functional decline in a cohort of older patients (mean age 79.4 years) who recovered from influenza pneumonia or acute respiratory illness, suggesting that COVID-19 impairs functional status more severely than other respiratory illnesses.

In our study, being autonomous and robust was a protective determinant of post-discharge functional autonomy, where almost seventy percent of participants losing ADL were frail or mildly disabled at hospital admission. These findings highlight the importance of the frailty assessment at hospital admission to promptly intercept trajectories towards poor outcomes, especially in those without pre-morbid disabilities.

Our study suffers from certain limitations. Firstly, caution must be applied with a small sample size, as the findings might not be transferable to all older COVID-19 patients during the first and second pandemic waves. Secondly, the limited statistical power might have impaired our ability to detect other significant predictors of poor outcomes. Thirdly, as an observational study, hidden bias and residual confounders might be operating. Fourthly, severe COVID-19 was less prevalent than in other case series of comparable age, and, consequently, the results of this study could underestimate the long-term effects of COVID-19 in frailest populations, such as nursing home residents. Finally, a comparison with age and sex-matched patients hospitalized without COVID-19 disease for the same period in identical centers would have been helpful.

In conclusion, the present study demonstrated that pre-morbid frailty status and disability outperformed age and COVID-19 severity as predictors of long-term adverse outcomes. Thus, a comprehensive geriatric assessment might help recognize high-risk individuals and promote periodic monitoring and rehabilitation programs, enhancing awareness among patients and clinicians regarding the post-acute phase of COVID-19. Given the high prevalence of newly diagnosed disability in our cohort of older adults, it could be reasonable to include functionality loss in the definition of “Long COVID-19 syndrome” in the case of patients with advanced age.

## Figures and Tables

**Table 1:** Clinical differences among survived and deceased patients.

	Whole Cohort (*n* = 193)	Alive (*n* = 150)	Dead (*n* = 43)	*p*-Value
Gender F (%)	84 (43.5)	58 (69.0)	26 (31)	0.011
Age mean (SD)	79.9 (9.1)	78.1 (8.7)	86.2 (7.7)	<0.001
ADL median (IQR)	6 (4)	6 (0.25)	2 (4)	<0.001
Frailty status Robust (%) Prefrail (%) Frail (%)	59 (30.6) 57 (29.5) 77 (39.9)	52 (34.7) 44 (29.3) 54 (36.0)	7 (16.3) 13 (30.2) 23 (53.5)	0.044
WHO status Mild disease (%) Moderate disease (%) Severe disease (%) Critical disease (%)	41 (21.2) 103 (53.4) 42(21.8) 7 (3.6)	35 (23.3) 79 (52.7) 29 (19.3) 7 (4.7)	6 (14.0) 24 (55.8) 13 (30.2) 0	0.15
NLR ratio baseline mean (SD)	8.8 (17.3)	9.2 (19.3)	7.6 (5.6)	0.61
Hs-CRP mean (n.v. < 5 mg/L)	77.3 (79.9)	75.1 (79.1)	82.2 (83.6)	0.67
Length of stay, median (days)	15 (15)	15 (11)	15.5 (19)	0.68
Number of comorbidities, median (IQR)	1 (2)	1 (3)	1 (2)	0.32
Arterial Hypertension (%)	129 (67.2)	101 (67.8)	28 (65.1)	0.74
Atrial fibrillation (%)	35 (18.2)	28 (18.8)	7 (16.3)	0.70
Heart failure (%)	34 (17.7)	28 (18.8)	6(13.9)	0.46
Diabetes type 1 or type 2 (%)	44 (22.9)	31 (20.8)	13(30.2)	0.19
Depression (%)	24 (12.4)	18 (12.0)	6 (14.0)	0.73
COPD (%)	32 (16.6)	26 (17.3)	6 (14.0)	0.81
Chronic Renal Failure (%)	32 (16.6)	25 (16.3)	7 (16.6)	0.95
Chronic Liver Failure (%)	5 (2.6)	5 (100)	0	0.22
Obesity (%)	16 (8.3)	12 (8)	4 (9.3)	0.78

ADL: activities of daily living; WHO: world health organization; NLR: neutrophils/lymphocytes ratio; Hs-CRP: High sensitivity C-reactive protein; COPD: chronic obstructive pulmonary disease; SD: standard deviation; IQR: interquartile range.

**Table 2:** Clinical features of patients losing or not ADLs.

	Whole Cohort (*n* = 132)	Without ADL Lost (*n* = 104)	With ADL Lost (*n* = 28)	*p*-Value
Gender F (%)	46 (34.8)	32 (69.6)	14 (30.4)	0.06
Age mean (SD)	77.4 (8.3)	76.2 (7.9)	81.8 (8.0)	0.001
ADL median (IQR)	6 (1)	6 (1)	6 (1.5)	0.56
Frailty status Robust (%) Prefrail (%) Frail (%)	49 (37.1) 40 (30.3) 43 (32.6)	48 (98) 32 (80) 24 (55.8)	1 (2) 8 (20) 19 (44.2)	<0.001
WHO status at admission Mild disease (%) Moderate disease (%) Severe disease (%) Critical disease (%)	32 (24.2) 86 (65.2) 4 (3.0) 10 (7.6)	27 (84.4) 64(74.4) 3 (75.0) 10 (100)	5(15.6) 22 (25.6) 1(25.0) 0(0)	0.23
P/F baseline mean (SD)	266 (104)	269 (109)	255 (84)	0.54
NLR (SD)	10.9 (22)	10.6 (22)	11.6 (21)	0.84
Hs-PCR (n.v. < 5 mg/L)	76 (77.1)	78.1 (84.2)	68.7 (49.1)	0.58
Length of stay, median (days)	14 (15)	14 (11)	20 (22)	0.038
Median Number of comorbidities (IQR)	1 (3)	1 (2)	2 (2)	0.036
Arterial Hypertension (%)	85 (64.4)	64 (61.5)	21 (75)	0.37
Atrial fibrillation (%)	25 (18.9)	19 (18.3)	6 (21.4)	0.78
Cardiac failure (%)	23 (17.4)	16(15.4)	7 (25)	0.23
Stroke (%)	12 (8.1)	11 (10.6)	1 (3.6)	0.46
Diabetes type 1 or type 2 (%)	28 (21.2)	20 (19.2)	8 (28.6)	0.28
Depression (%)	15 (11.4)	8 (7.7)	7 (25)	0.010
COPD (%)	12 (9.1)	9 (8.7)	3 (10.7)	0.73
Chronic Renal Failure (%)	16 (16.9)	11 (10.6)	5 (17.9)	0.29
Chronic Liver Disease (%)	1 (0.8)	1(100)	0 (0)	0.60
Obesity (%)	8 (6.1)	6 (5.78)	2 (7.1)	0.78

ADL: activities of daily living; WHO: world health organization; NLR: neutrophils/lymphocytes ratio; Hs-CRP: High sensitivity C-reactive protein; COPD: chronic obstructive pulmonary disease; SD: standard deviation; IQR: interquartile range.

## Data Availability

Restrictions apply to the availability of these data. Data was obtained from the GeroCovid Observational Board and are available from the authors with the permission of the GeroCovid Observational Board.

## Supplementary Materials

The following supporting information can be downloaded at: https://www.mdpi.com/article/10.3390/jcm11195578/s1, Figure S1: Six-month all-cause mortality. Kaplan Meier curve; Table S1: 30-items Frailty Index; Table S2: Six-month discharge cause-specific mortality; Table S3: Correlation matrix between lost ADL at six-month follow-up; Table S4: Drug therapy received by the study population during hospitalization.
