# Supplementary material for: Determinants of Cause-Specific Mortality and Loss of Independence in Older Patients following Hospitalization for COVID-19: The GeroCovid Outcomes Study
Source: J Clin Med. 2022 Sep 22;11(19):5578. doi: 10.3390/jcm11195578 (PMC9571114; doi:10.3390/jcm11195578)
Supplement: Supplementary file 1 [file jcm-11-05578-s001.zip › jcm-1845385-supplementary.pdf]

Figure S1: Six-month all-cause mortality. Kaplan Meier curve**.**

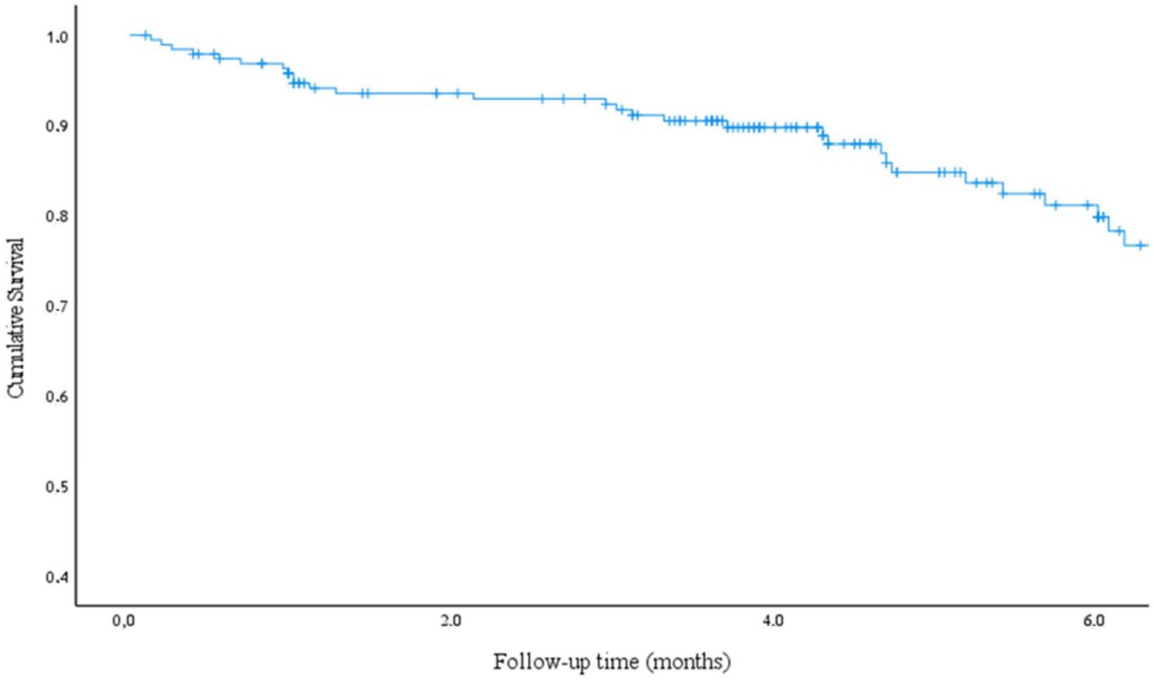

Table S1: 30-items Frailty Index

FRAILTY INDEX 30 ITEMS	0 POINT	0.5 POINT	1 POINT
**Bathing**	Preserved	Impaired	Lost
**Dressing**	Preserved	Impaired	Lost
**Toileting**	Preserved	Impaired	Lost
**Transferring**	Preserved	Impaired	Lost
**Continence**	Preserved	Impaired	Lost
**Feeding**	Preserved	Impaired	Lost
**Ability to Use Telephone**	Preserved	Impaired	Lost
**Shopping**	Preserved	Impaired	Lost
**Food Preparation**	Preserved	Impaired	Lost
**Housekeeping**	Preserved	Impaired	Lost
**Laundry**	Preserved	Impaired	Lost
**Mode of Transportation**	Preserved	Impaired	Lost
**Responsability for Own Medications**	Preserved	Impaired	Lost
**Ability to Handle Finances**	Preserved	Impaired	Lost
**Mobility Function**	Independent	With Aid	Dependent
**Obesity**	No	-	Yes
**Poor nutritional Status**	No	Suspect	Yes
**Number of Medication**	0-3	4-6	≥ 7
**Co-habitation Status**	Living with Family	Institutionalized	Living Alone
**History of Depression**	No	Suspect	Yes
**History of Cognitive Impairment**	No	Suspect	Yes
**History of Hypertension**	No	Suspect	Yes
**History of Heart Failure**	No	Suspect	Yes
**History of Ischemic Heart Disease**	No	Suspect	Yes
**History of Diabetes**	No	Suspect	Yes
**History of Stroke**	No	Suspect	Yes
**History of Chronic Renal Failure**	No	Suspect	Yes
**History of Chronic Liver Failure**	No	Suspect	Yes
**History of COPD**	No	Suspect	Yes
**History of Cancer**	No	Suspect	Yes
	**TOTAL SCORE**	**/30 =**	

Table S2: Six-month discharge cause-specific mortality

**Cardiovascular Diseases (n = 19)**	Cardiac arrest (5)<br>Acute Heart Failure (11)<br>Acute myocardial injury (2)<br>Unspecified (1)
**Respiratory Failure (n=11)**	Acute respiratory failure (11)
**Genitourinary Disorders (n = 3)**	Urinary tract infections (3)
**Cancer (n = 2)**	Rectal cancer (1)<br>Gastric cancer (1)
**Other causes (n = 7)**	Stroke (1)<br>Hypovolemic shock (2)<br>Septic shock (3)<br>Acute hepatitis (1)<br>Unspecified (1)

Table S3: Correlation matrix between lost ADL at six-month follow-up

	BATHING	DRESSING	TOILETING	TRANSFERRING	CONTINENCE	FEEDING
BATHING		0.902	0.872	0.872	0.772	0.688
DRESSING	0.902		0.866	0.866	0.798	0.712
TOILETING	0.872	0.866		0.890	0.850	0.792
TRANSFERRING	0.872	0.866	0.890		0.850	0.652
CONTINENCE	0.772	0.798	0.850	0.850		0.772
FEEDING	0.688	0.712	0.792	0.652	0.772	
	Low-to-moderate correlation (0.30 to 0.50)					
	Moderate-to-high correlation (0.50-70)					
	Very strong correlation (0.70-1.00)					

Table S4: Drug therapy received by the study population during hospitalization.

	All patients N=193	Alive N=150	Dead N=43	P-value
Antibiotics (%)	113(60.4)	87(60.0)	26(61.9)	0.82
Antiviral therapy (%)	47(25.1)	34(23.4)	13(31.0)	0.32
LWMH (%)	127(67.9)	100(69.0)	27(64.3)	0.56
Immuno-modulators (%)	9(4.8)	7(4.8)	2(4.8)	0.98
Corticosteroids (%)	89(47.6)	65(44.8)	24(57.1)	0.16
Hydroxychloroquine (%)	62(33.2)	46(31.7)	16(38.1)	0.44
